# Methylprednisolone Therapy in Acute Hemorrhagic Edema of Infancy

**DOI:** 10.1155/2014/853038

**Published:** 2014-03-05

**Authors:** Jeyanthini Risikesan, Uffe Koppelhus, Torben Steiniche, Mette Deleuran, Troels Herlin

**Affiliations:** ^1^Department of Pediatrics, Aarhus University Hospital, 8200 Aarhus N, Denmark; ^2^Department of Dermatology, Aarhus University Hospital, Aarhus C, 8000 Aarhus, Denmark; ^3^Department of Pathology, Aarhus University Hospital, Aarhus C, 8000 Aarhus, Denmark

## Abstract

We present a case of an 18-month-old boy who showed severe clinical signs indicative of acute hemorrhagic edema of infancy (AHEI) with painful purpuric skin affection primarily of the face and marked edema of the ears. The histological findings were diagnostic for leukocytoclastic vasculitis and thus met the histological criteria for AHEI. Indicative of infection as causative agent for the condition were symptoms of gastroenteritis. High-dose intravenous corticosteroids led to a fast resolution of symptoms and normalization of laboratory parameters. AHEI is usually not described as being very responsive to corticosteroids. The case presented here indicates that severe cases of AHEI can be treated with high-dose intravenous corticosteroids resulting in significant relief and shortening of the symptoms. Clinical followup showed no underlying malignancy or other severe chronic systemic diseases thus confirming earlier reports that AHEI is not associated with such conditions. The differential diagnoses with AHEI are discussed.

## 1. Introduction

Acute hemorrhagic edema of infancy (AHEI) is an uncommon benign form of cutaneous small-vessel leukocytoclastic vasculitis, which typically affects children from 4 to 24 months of age [1]. AHEI was first described by Snow in 1913 [2]. A case series and systematic review by Fiore et al. [3] has reported approximately 300 patients with AHEI, with a male predominance. The pathogenesis is not fully understood, but a prodromal phase with various infections has been documented in children with AHEI. These include upper respiratory infections, pharyngitis, conjunctivitis, otitis media, bronchitis, urinary tract infections, and pneumonia [4].

AHEI is characterized by the clinical triad of fever; edema of the face, auricles, and extremities; and rosette-shaped purpura. Unlike Henoch-Schönlein purpura (HSP) visceral involvement is infrequent [1]. A correct diagnosis of the disorder is important to distinguish it from other vasculitides. The clinical features of AHEI may be confused with the symptoms seen in (HSP), erythema multiforme (EM), meningococcemia, and septicemia. The diagnostic criteria for AHEI are (1) age younger than 2 years; (2) purpuric or ecchymotic “bruise-like” skin lesions with edema of the face, auricles, and extremities with or without mucosal involvement; (3) lack of systemic disease or visceral involvement and spontaneous recovery within a few days or weeks [5].

AHEI is self-limiting (lasting from one to three weeks) and is not usually considered responsive to corticosteroids [1]. We present a severe case of AHEI in an 18-months-old boy who responded rapidly to high-dose systemic corticosteroids.

## 2. Case Report

**Figure 1:**
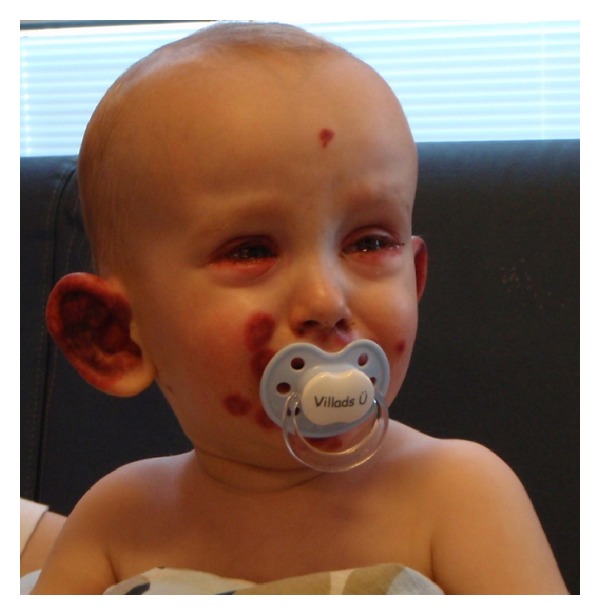
Purpura lesions distributed over the face and both ears in an 18-month-old boy. The ears were edematous and had a bright-red, nearly purple color.

**Figure 2:**
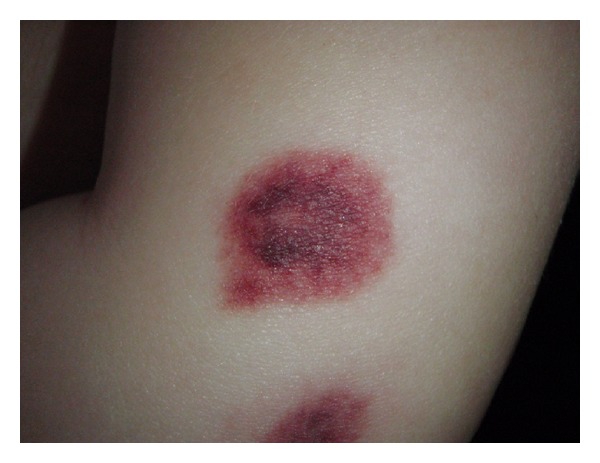
Tender, bright-red, infiltrated papules and nodules were found on the extremities and trunk.

**Figure 3:**
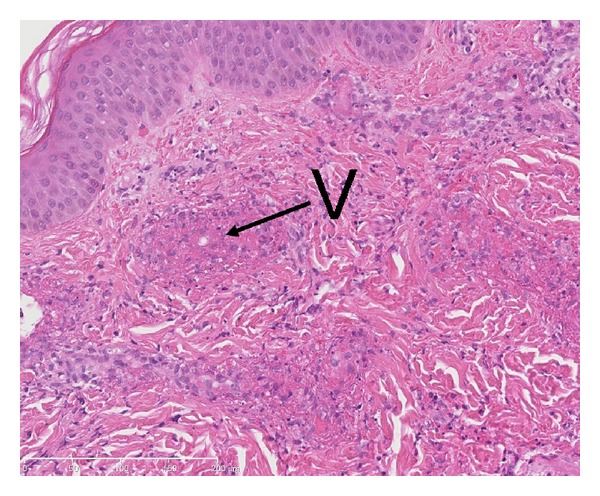
Biopsy from cutaneous lesion showed a neutrophilic infiltrate and exudation of fibrin (fibrinoid necrosis) in the walls of small vessels (V) and in their vicinity concordant with leukocytoclastic vasculitis.

An 18-month-old boy was admitted to the pediatric department with fever and rash. Prior to admission he had a four-day history of gastroenteritis, fever, and incipient skin eruption on the ear and purpuric elements on the extremities. On suspicion of meningococcal disease lumbar puncture was performed which revealed normal spinal fluid. Treatment with ceftriaxone was started. The skin lesions progressed, especially in the perioral area, in numbers and size and became bright-red and circular. Moreover, they became increasingly painful for the patient. Also, periorbital blushing was observed. The ears were edematous and had a bright-red, nearly purple color (Figure 1). Furthermore, tender, bright-red, slightly infiltrated papules and nodules were found on the extremities and trunk (Figure 2). The skin was otherwise intact. Temperature was 38.2°C. C-reactive protein (CRP) increased to max. of 135.9 mg/L and erythrocyte sedimentation rate (ESR) to 38 mm/hr. Leukocyte and platelet counts were within normal range. Skin biopsy was performed and high-dose corticosteroid treatment with intravenous (i.v.) methylprednisolone 20 mg/kg/day for 4 days was given. Histology of skin lesion was described as a well-established leukocytoclastic vasculitis with marked fibrinoid necrosis and granulocyte infiltration in the vessel wall (Figure 3). The treatment resulted in immediate declining of the edema and bleaching of the skin lesions and normalization of laboratory parameters. Over the next days a general improvement of the skin symptoms was seen even though a few new skin lesions appeared. Following pulse-steroid treatment with oral prednisolone, 2 mg/kg/day, was continued. Ten days after treatment was started he was readmitted with relapse of fever and relapse of an ecchymosis and several palpable purpura on both lower legs. Pulse-steroid treatment was repeated for 3 days, and all symptoms improved within two days. Prednisolone was then continued and tapered over 12 days. Clinical followup during the next 30 months did not show any episodes of relapse, and the patient appears completely well without any signs of sequelae.

## 3. Discussion

We present a case of an 18-month-old boy with severe vasculitis-like affection primarily of the face and marked edema of the ears. He had a prodromal period with gastroenteritis and the histological findings were diagnostic for leukocytoclastic vasculitis. The clinical findings met the criteria for the diagnosis of AHEI. Prompt recognition of this rare disease is important to differentiate it from other manifestations that require specific therapy. The main differential diagnosis of AHEI of young children includes HSP [6, 7]. In Table 1 the main characteristics and differences between the two diseases are outlined.

As seen from Table 1, there is no internal organ involvement in AHEI. The cutaneous findings are dramatic, both in appearance and rapidity of onset, and may therefore cause significant anxiety for parents as well as clinicians. Both AHEI and HSP are leukocytoclastic vasculitides, but the immunohistology in AHEI is different from the pattern of HSP. In AHEI there is more extensive vasculitis with fibrin deposits; IgA deposits are seen in a minority of cases. The target lesions of erythema multiforme (EM) often first appear over the dorsum of the hands progressing centripetally to involve the proximal extremities and the trunk. The severe form of erythema multiforme, Stevens-Johnson syndrome, may have hemorrhagic and papular lesions resembling AHEI, but it includes ulcerating lesions of the mucous membranes. Other differential diagnoses of AHEI include meningococcal sepsis, purpura fulminans, eruptions of viral infections, drug-induced vasculitis, and Sweet's syndrome [8]. All these disorders can be differentiated from AHEI by results of history, physical examination, and appropriate laboratory studies, including histological examination of a skin biopsy. There is no specific treatment for patients with AHEI. In a recent review, corticosteroids and antihistamines were not reported to alter the course of the disease [8]. However, few publications have reported beneficial effect of steroids in AHEI [9], and nonsteroidal anti-inflammatory drugs are recommended for tender skin lesions or in cases with musculoskeletal pain [8, 9]. Furthermore, antibiotics are indicated when bacterial infection is suspected [10].

In the case reported here a clear improvement of the disease was seen immediately after high-dose therapy with i.v. methylprednisolone was started. However, after lowering the corticosteroid dose a relapse was observed but rapid improvement was obtained after repeating the high-dose i.v. methylprednisolone. Our results suggest that in severe cases of AHEI high-dose corticosteroids should be considered.

## Figures and Tables

**Table 1:** Clinical differences between acute hemorrhagic edema of infancy (AHEI) and Henoch-Schönleins purpura (HSP).

Clinical findings	AHEI	HSP
Peak incidence	4 to 24 months	4 to 7 years
Skin distribution	Faces, auricles, and extremities	Extensor surfaces of the legs and buttocks
Edema	Consistent, nonpitting	Inconsistent
Gastrointestinal involvement	Rare	Common
Articular involvement	Rare	Common
Renal involvement	Extremely rare	Common
Skin histology	Leukocytoclastic vasculitis, frequently with fibrinoid necrosis	Leukocytoclastic vasculitis
Perivascular deposits	C1q	IgA
Duration	2-3 weeks	1 month or more
Relapses	Rare	Frequent
